# Immunoregulatory orchestrations in osteoarthritis and mesenchymal stromal cells for therapy

**DOI:** 10.1016/j.jot.2025.08.009

**Published:** 2025-08-26

**Authors:** Tongmeng Jiang, Shibo Su, Ruijiao Tian, Yang Jiao, Shudan Zheng, Tianyi Liu, Yang Yu, Pengbing Hua, Xiuhong Cao, Yanlong Xing, Panli Ni, Rui Wang, Fabiao Yu, Juan Wang

**Affiliations:** a Key Laboratory of Tropical Translational Medicine of Ministry of Education & Key Laboratory of Brain Science Research and Transformation in Tropical Environment of Hainan Province, Hainan Provincial Stem Cell Research Institute, School of Basic Medicine and Life Sciences, Hainan Medical University, Haikou, 571199, China; b Engineering Research Center for Hainan Bio-Smart Materials and Bio-Medical Devices, Key Laboratory of Hainan Functional Materials and Molecular Imaging, College of Emergency and Trauma, Hainan Academy of Medical Sciences, Hainan Medical University, Haikou, 571199, China; c Key Laboratory of Emergency and Trauma of Ministry of Education, Key Laboratory of Haikou Trauma, Key Laboratory of Hainan Trauma and Disaster Rescue, The First Affiliated Hospital, Hainan Medical University, Haikou, 571199, China; d Department of Biomedical Engineering, Faculty of Engineering, The Hong Kong Polytechnic University, Hong Kong, China; e Plastic Surgery, Second Affiliated Hospital of Hainan Medical University, Haikou, 570100, China; f Department of Pharmacology, Zibo Hospital of Traditional Chinese Medicine, Zibo, 255300, China; g The Second Department of Urology, The Second Affiliated Hospital of Dalian Medical University, Dalian, 116021, China

**Keywords:** Immunomodulation, Macrophage, Mesenchymal stromal cells, Osteoarthritis, T cells

## Abstract

Osteoarthritis (OA) is characterized by the inability of stable and complex joint structures to function as they did, accompanied by inflammation, tissue changes, chronic pain, and neuropathic inflammation. In the past, the primary focus on the causes of joint dysfunction has been on mechanical stress leading to cartilage wear. Further researches emphasize the aging of cartilage and subchondral bone triggered cartilage lesion and osteophyte formation. Recently, the effects of immune cells, particularly macrophages and T cells, have been receiving focused attention. Herein, we primarily discuss the role of macrophages and T cells in the progression of OA and how mild inflammation in cartilage, subchondral bone, synovium, muscles, and nerves influences the progression of OA. Additionally, this review highlights the interaction between mesenchymal stromal cells (MSCs) and macrophages, as well as MSCs and T cells, along with how these interactions affect OA development and treatment. Finally, we explore future research directions and issues that still need to be addressed, providing more insights for the clinical translation of MSC-based therapy for OA.

**The translational potential of this article:**

This review highlights the promising translational potential of MSCs in OA therapy by targeting immunoregulatory networks. MSCs directly modulating macrophage M1/M2 polarization, Th1/Th2 and Th/Treg balance of T cells to suppress inflammation, thereby promoting cartilage repair and subchondral bone remodeling. Their ability to synergize with biomaterials or drug carriers enhances therapeutic precision and efficacy. However, challenges like MSCs survival in inflammatory microenvironments, heterogeneity in immune cell responses, and personalized treatment strategies require further optimization. Advances in genetical engineered strategies, extracellular vesicles, scaffolds/hydrogels or nanoparticle-based approaches may bridge these gaps, offering scalable solutions for clinical translation. This work underscores MSC-based therapies as a transformative approach for OA, pending refinement of delivery systems and patient stratification.

## Graphical abstract

**Figure:**
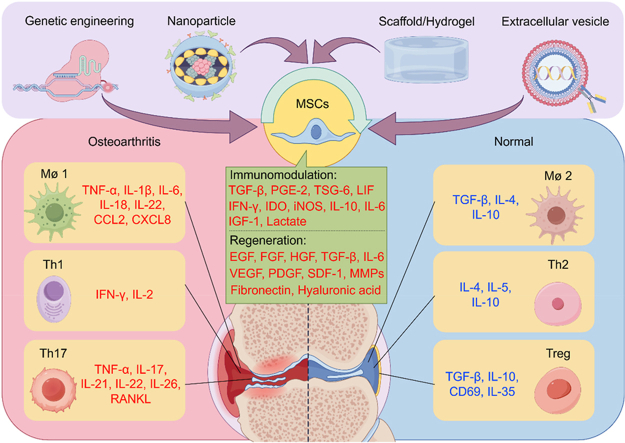

## 1 Introduction

**Fig. 1:**
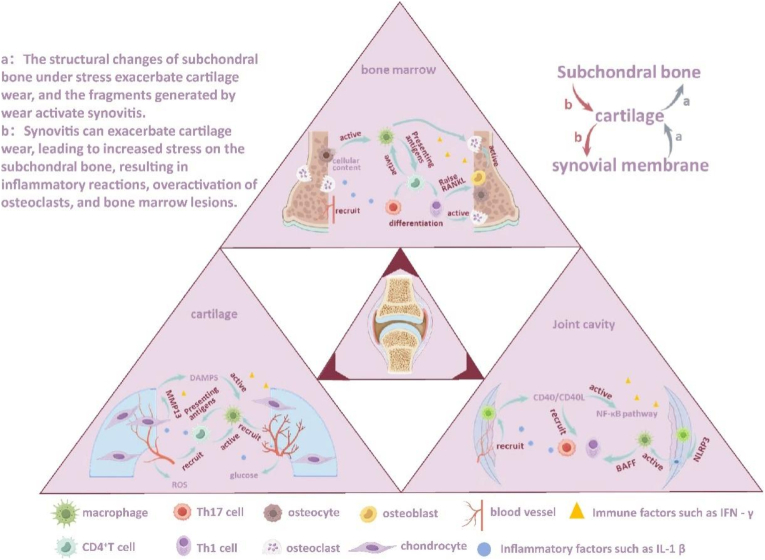
**The immune microenvironment in the development of OA.** Mechanical stress triggers the macrophage M1 polarization and CD4^+^ T cell differentiation, thereby accelerating the inflammation of osteocytes, chondrocytes and synovial cells in subchondral bone, cartilage and synovial membrane respectively.

Osteoarthritis (OA) is a widespread joint disease where molecular changes in joint tissue lead to pathological cartilage remodeling, osteophyte formation, synovitis, and damage to adjacent soft tissues, ultimately causing pain and impaired joint function, with profound impacts on individuals and society [1]. In the early stages of OA, joint cartilage wears out due to frequent pressure and friction. However, the lubricating substances secreted by synovial tissue, along with the coordinated actions of muscles, tendons, and ligaments, keep the wear minimal and do not affect the normal function of joint cartilage. Nevertheless, the debris from wear can transform into damage-associated molecular patterns (DAMPs) signals, activating macrophages and promoting their polarization towards M1, which in turn enhances the secretion of inflammatory factors, shaping an inflammatory microenvironment [2,3]. After this initial inflammation of OA is triggered by macrophages polarizing towards M1, more pro-inflammatory cytokines and activate immune system mediators occur to create an inflammatory microenvironment in the joint cavity later. This environment affects the activity of chondrocytes, synovial cells, and osteocytes, thereby exacerbating OA [4](Fig. 1). During the progression of OA, the immune regulation they mediate may be essential for the body but must remain within controllable limits. The negative feedback loops formed between various structures during the development of OA do not keep inflammation under control for long periods. Additionally, besides macrophages, there are T cells, B cells, mast cells, and NK cells within the synovial tissue, indicating that OA involves intricate immune system regulation [5]. Recently, the roles of T cells on OA have been attracted many of practitioners [6], especially CD4^+^ T cells in modulating the inflammation process [7]. Among the CD4^+^ T cells, the balance between Th1/Th2 and Th17/Treg are most important in the development of OA. In particular, Th1 and Th17 cells promote the progression of OA, whereas Th2 and Treg prevent OA from becoming worse [6]. Thus, modulation the immune microenvironment in the joint may a new strategy for OA therapy. Mesenchymal stromal/stem cells (MSCs) are endogenous inflammatory modulators widely but minimally present in various tissues. When these tissues are exposed to an inflammatory microenvironment, MSCs release various mediators to coordinate local innate and adaptive immune responses, inhibit typical inflammatory behaviors of target cells, and promote the production of anti-inflammatory cells [8,9]. However, due to the limited number of endogenous MSCs and the complexity of the repair process, this endogenous anti-inflammatory behavior mediated by MSCs cannot rapidly and efficiently repair tissues or improve the inflammatory microenvironment. Therefore, the transplantation of MSCs for disease treatment has gradually matured and developed rapidly [10].

**Fig. 2:**
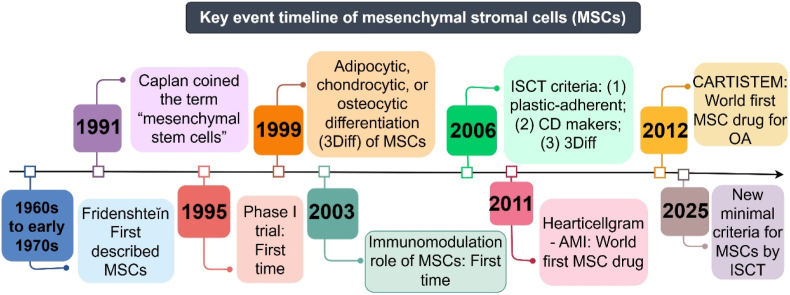
Key event timeline of MSCs for OA therapy. ISCT: International Society for Cell & Gene Therapy. By Figdraw.

MSCs were first identified by Friedenstein in late 1960s to early 1970s [11], and coined the term “Mesenchymal stem cells” by Caplan in 1991 [12]. A first Phase Ⅰ clinical trial of MSCs has been launched at 1995 and declared the safety of MSCs applied in human body [13]. In 1999, a reputative journal “Science” reported the three-differentiation (3Diff) potential, including adipocytic, chondrocytic and osteocytic of human derived MSCs [14], which has been referred to the three minimal criteria for identifying MSCs indicated by International Society for Cell & Gene Therapy (ISCT) in 2006 [15]. However, this criterion has been deleted by ISCT in 2025 with the plastic adherent phenotype, while only the CD marker identifications in the former three minimal criteria remained [16]. More importantly, ISCT prefers the term “Mesenchymal stromal cells” rather than “Mesenchymal stem cells” of MSCs [16]. The immunomodulation role of MSCs were first indicated by Le Blanc K et al. [[17], [18], [19]], thereby a new era of MSCs for immunomodulation began. The world's first MSC drug Hearticellgram^Ⓡ^-AMI is constructed by bone marrow-derived MSCs (BMSCs) and used to treat patients with acute myocardial infarction in 2011 [20]. And the world's first MSC drug CARTISTEM® for OA therapy is constructed by human Umbilical Cord Blood-derived MSCs (UC-MSCs) to treat OA with cartilage defect at International Cartilage Repair Society (ICRS) grade IV [21](Fig. 2). Therefore, transplantation of exogenous MSCs are welcome for orthopaedic surgeons in the field of OA therapy [22].

Exogenous MSCs can come from autologous or allogeneic sources, similar to endogenous MSCs, they possess immune regulatory capabilities and multi-directional differentiation potential with regenerative effects [23]. In the mild inflammation stage, endogenous MSCs are sufficient to respond to the inflammatory response and play an immune regulatory role to inhibit inflammation [24]. However, in the severe inflammation stage, it is necessary to rely on exogenous stem cells to work together with endogenous stem cells to play an immune regulatory role and then inhibit inflammation [25,26]. At present, the research of MSCs in the treatment of OA has been relatively deep. Fat, bone marrow and umbilical cord-derived MSCs can significantly repair cartilage defects, promote cartilage matrix repair, and regulate the inflammatory microenvironment and oxidative stress microenvironment in the joint cavity [27]. Moreover, MSCs can also exert immune regulatory effects by regulating the phenotypic transition of macrophages [28]. However, as mentioned above, the factors that contribute to and maintain OA are relatively complex, and the immune regulatory mechanisms involved have not been clarified in detail. How MSCs associate with CD4+T cells in the process of exerting immune regulation remains to be further explored. Moreover, they can also be implanted in larger quantities and can be combined with drugs or scaffold materials, thus offering greater controllability and higher efficacy [29,30].

**Fig. 3:**
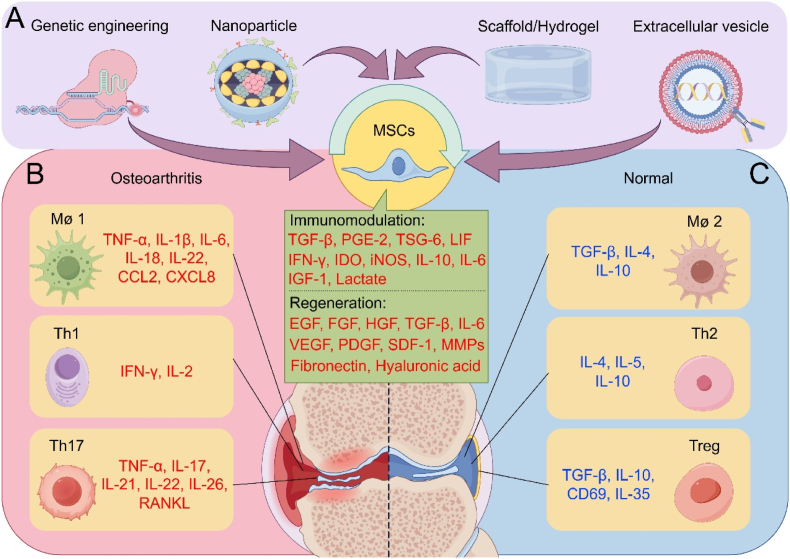
**MSCs modulate the balances of M1/M2 macrophage, Th1/Th2 and Th17/Treg for OA treatment.** (A) MSCs secrete different factors for immunomodulation and regeneration in OA therapy. Genetical engineering, nanoparticles, hydrogel/scaffolds and extracellular vesicles are also promising approaches for MSC modification and application. (B) M1 macrophage, Th1, Th17 cells and their secret factors stimulate OA. (C) M2 macrophage, Th2, Treg cells and their secret factors maintain the joint in the normal type. By Figdraw. IL: interleukin; TNF-α: tumor necrosis factor-α; TSG-6: TNF stimulated gene-6; IFN-γ: interferon-γ; CCL2: C–C motif chemokine ligand 2; CXCL8 (also known as IL-8): C-X-C motif chemokine ligand 8; TGF-β: transforming growth factor-β, PGE-2: prostaglandin E2; LIF: LIF interleukin 6 family cytokine; IDO: indoleamine; iNOS: inducible nitric oxide synthase; IGF-1: insulin like growth factor-1; EGF: epidermal growth factor; FGF: fibroblast growth factor; HGF: hepatocyte growth factor; VEGF: vascular endothelial growth factor; PDGF: platelet derived growth factor; SDF-1: stromal cell-derived factor-1; MMPs: matrix metalloproteinases; RANKL: receptor activator of nuclear factor-κ B Ligand.

To fill the gap, this paper discusses the intricate role of MSCs in regulating the immune balances of M1/M2 macrophage, Th1/Th2 and Th17/Treg in OA development, as well as how MSCs would prevent OA progression. The cutting-edge technology for modifying MSCs including genetical engineering, nanoparticle application, hydrogel/scaffolds and extracellular vesicles as promising approaches for OA therapy are also introduced (Fig. 3). The aim is to enhance understanding of the immune mechanisms involved in pathogenesis the treatment of OA, thereby offering new insights for the development of MSC-based therapeutics, relevant biomaterials and the selection of appropriate drugs.

## 2 Complex regulation in the occurrence and development of OA

### 2.1 Joint development process and OA

Articular cartilage is the transparent cartilage tissue that covers the ends of bone structures, primarily composed of water and extracellular matrix (ECM) of chondrocytes, accounting for 95 % of its total volume. The remaining 5 % consists of chondrocytes [31]. Due to its unique developmental pattern, it does not contain blood vessels, lymphatic vessels, or nerves, which results in chondrocytes being in a low-oxygen, low-nutrient environment and renewing the components of the ECM at a low turnover rate. As mentioned earlier, pressure can lead to cartilage wear, and due to the lack of lymphatic vessels, the clearance of debris from wear can only rely on mononuclear macrophages residing in the joint cavity and synovial lining, but this also promotes the formation of an inflammatory microenvironment [2]. On the other hand, wear can trigger the aging and abnormal death of chondrocytes [3], leading to mitochondrial dysfunction within the cells, which in turn fosters an oxidative stress microenvironment [3,32]. In addition, these mechanical changes also lead to the abnormal cartilage calcification in the development of OA [33].

**Fig. 4:**
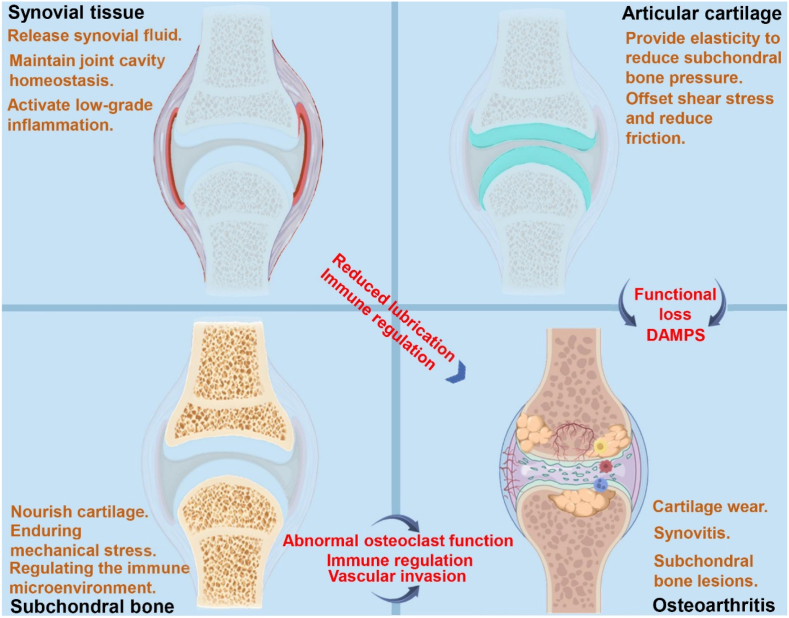
**The pathological process of synovial tissue, articular cartilage and subchondral bone during OA.** DAMPs: damage associated molecular patterns.

Due to the close and intimate relationship between subchondral bone and hyaline cartilage, the inflammatory microenvironment of hyaline cartilage to some extent also promotes the inflammatory microenvironment of subchondral bone. The relationship among articular cartilage, synovial tissue and subchondral bone in OA is shown in Fig. 4.

Subchondral bone refers to the calcified layer beneath hyaline cartilage, which contains nerve fibers and blood vessels, responsible for providing mechanical support and nutrition to hyaline cartilage [34]. Healthy subchondral bone automatically adjusts the concentration and direction of channels within trabeculae when subjected to mechanical stress, adapting to multidimensional and frequently changing stresses. However, if the burden of mechanical stress increases, such as when hyaline cartilage no longer maintains its elasticity, subchondral bone may begin to develop microfractures, which is the cause of bone marrow lesions [35]. If the reasons for abnormal loading persist or bone marrow lesions continue to worsen, subchondral bone will undergo abnormal bone remodeling, pathologically characterized by vascular infiltration, increased bone mass but low mineral density [36]. As vascular infiltration and fissures occur, synovial fluid can penetrate, and blood vessels serve as channels for inflammatory factors. Due to the influence of the inflammatory microenvironment, osteoblast differentiation and maturation are significantly inhibited, while osteoclastogenesis mediated by osteoblasts increases, along with the proliferation and differentiation of osteoclasts. These factors exacerbate further calcium loss in bones and maintain abnormal mechanical loads to a great extent, forming a vicious cycle [37]. Therefore, influenced by the inflammatory microenvironment of hyaline cartilage, the subchondral bone also develops an inflammatory microenvironment, and the two mutually affect each other, further intensifying the progression of inflammation. Currently, clinical treatments for OA include oral nonsteroidal anti-inflammatory drugs, intra-articular hyaluronic acid injections, and surgical interventions. Nonsteroidal anti-inflammatory drugs primarily aim to relieve pain, with their effectiveness in suppressing inflammation varying from person to person. Additionally, these medications inevitably come with side effects and the risk of drug resistance. Surgical options are also costly, and postoperative care and mobility restrictions are significant concerns [38,39]. In short, none of the current treatment methods can effectively address OA.

### 2.2 Aging and obesity aggravate the inflammation of OA

OA was once classified as a non-infectious "non-inflammatory" joint disease, but in fact, its inflammatory response is highly complex and involves tissues such as the synovium, subchondral bone, synovial tissue, and the patellar fat pad [40]. The occurrence of this inflammation is believed to be the result of innate immune system activity, which seeks to restore tissue homeostasis in response to infection or injury [41]. After the body is exposed to harmful stimuli, the first step is to detect these stimuli through pattern recognition receptors on cell surfaces. These receptors include pathogen-associated molecular patterns (PAMPs), which activate lineage-encoded pattern recognition receptors (PRRs) in both immune and non-immune cells. DAMPs are also recognized by PRRs, responding to signals released during tissue or cell damage. Several intracellular signaling pathways, including nuclear factor-κB (NF-κB) and mitogen-activated protein kinase (MAPK), are activated after receptor activation. The activation of inflammatory cells, such as macrophages and adipocytes, triggers the release of inflammatory markers, including various cytokines (leukocyte interleukins, colony-stimulating factors, IFN, TNF, TGF, and chemokines). Additionally, damaged cells release cytokines known as "alarmines," such as interleukin-1α (IL-1α) and interleukin-33 (IL-33), which induce the migration of immune cells. DAMPs also induce the activation of immune cells [2,38,42]. Precise coordination between macrophages and tissue-specific cells is crucial for restoring damaged tissues and maintaining homeostasis within the body [43].

The synovium is one of the primary sites of inflammation in OA. The synovial tissue consists of two layers of cells: the lining layer, composed of fibroblast-like synoviocytes and macrophages, and the sublining layer, made up of vascularized connective tissue [44]. The lining layer is mainly composed of synovial-like fibroblasts and macrophages, while the sublining layer comprises ECM, blood vessels, fibroblasts, macrophages, and fat cells [45]. The presence of synovial tissue ensures adequate lubrication of the joint surface, which primarily depends on the secretion of synovial fluid. The main components of synovial fluid are plasma ultrafiltrate and hyaluronic acid and lubricin secreted by synovial-like fibroblasts. The former relies on relatively normal vascular permeability and blood flow, while the latter depends on the ability of synovial-like fibroblasts to function normally [46]. Notably, inflammatory mediators IL-1β and TNF-α are secreted early in OA, followed by TNF-α stimulating TNF receptor 1 (TNFR1) and TNF receptor 2 (TNFR2). Both receptors are expressed in the synovium, with TNFR1 strongly inducing pro-inflammatory effects and TNFR2 capable of mediating both pro-inflammatory and anti-inflammatory responses based on pathology. These pro-inflammatory factors stimulate the production of large amounts of nitric oxide (NO) through inducible nitric oxide synthase (iNOS), thereby increasing the synthesis of prostaglandin E2 (PGE2) and cyclooxygenase (COX2). At the same time, PGE2 increases the production of MMP13, leading to collagen degradation [[47], [48], [49]].

Aging is another risk factor for OA, difficult to avoid and plays a significant role in its development [50]. Comparing inflammatory markers in the lungs of mice at different ages, it was found that the levels of inflammatory markers in the lungs of aged mice were significantly higher, indicating a connection between senescent cells and inflammation [51]. Obesity is also a major cause of OA. Due to excessive joint load and mild systemic inflammation, it affects both weight-bearing and non-weight-bearing joints. This systemic inflammation is caused by pro-inflammatory adipokines secreted by cells from adipose tissues around or with the joints, such as subpatellar fat pads and alar folds [52]. Most importantly, fat also adheres to immune cells like T-cells and macrophages. For example, in the bone marrow, adipose tissue provides space for macrophages to adhere and proliferate, thereby inducing a strong inflammatory immune response when activated by specific antigens. Additionally, obese patients have a higher incidence and severity of synovitis, characterized by synovial fibrosis, increased macrophage infiltration, and elevated Toll-like receptor-4 (TLR4) expression. Although obesity significantly promotes synovitis, synovitis does not seem to improve after weight loss [8,53]. Moreover, diabetes can also lead to OA, especially type 2 diabetes, as these patients typically exhibit high body weight phenotypes, which increase joint load. High glucose levels also have a significant impact, providing the necessary energy for immune cell proliferation and differentiation and triggering oxidative stress [54].

### 2.3 Immune changes in the development of OA

OA progression is driven by profound immune dysregulation across joint tissues. Synovitis features macrophage polarization imbalance and T-cell subset dysregulation, amplifying pro-inflammatory cytokines that degrade cartilage. In subchondral bone, osteoclast hyperactivity—triggered by inflammatory cues and macrophage-derived RANKL—induces pathological bone loss and vascular invasion, disrupting cartilage homeostasis. Synergistically, oxidative stress from neovascularization and immune cell infiltration accelerates chondrocyte apoptosis and ECM breakdown. These interconnected immune alterations establish a self-perpetuating cycle of joint destruction. This section integrates key mechanisms including synovial inflammation, osteoclast-mediated bone remodeling, and immune cell crosstalk in cartilage, aligning with the section's focus on OA pathogenesis driven by immunity.

#### 2.3.1 Inflammation in synovium and its immune regulation

As aforementioned, synovial hypertrophy and synovitis are clear indicators of OA. The synovium comprises two distinct layers: the intima (synovial lining layer) and the subintima (sublining layer). The lining layer is mainly composed of synovial fibroblasts and macrophages, while the sublining layer is composed of the ECM, blood vessels, fibroblasts, macrophages and adipocytes [55]. The presence of synovial tissue ensures adequate lubrication of the joint contact surfaces, which relies mainly on that synovial tissue can secrete synovial fluid. The main components of synovial fluid are plasma ultrafiltrate and hyaluronic acid secreted by synovial-like fibroblasts, which rely on relatively normal vascular penetration and blood flow, while the latter depends on synovial-like fibroblasts that can perform normal function [55].

Regarding the causes of synovitis, current research has not yet determined its priority in the pathogenesis of OA. While the precise etiological relationship between synovitis and OA remains to be fully elucidated, the aberrant polarization of macrophages within the synovial lining undoubtedly plays a critical role in the pathogenesis of the disease. Inflammatory synovial tissue exhibits a marked increase in synovial lining macrophage populations, representing a substantial expansion of the total macrophage count [56]. Such a large number of macrophages, on the one hand, originate from the massive proliferation of existing macrophages away from the resting state, and from the CD80/CD86 surface factor overexpressed after macrophage polarization, in addition to recall and activate macrophages in peripheral blood, they also stimulate the activation and survival of CD4^+^ T cells [57,58]. The observed increase in macrophage population is consistent with a typical inflammatory response, given their roles in antigen presentation, CD4^+^ T cell activation, and phagocytosis [59]. However, impaired macrophage phagocytosis coupled with the sustained overexpression and release of inflammatory factors presents a detrimental scenario for inflamed tissues [60]. Hu et al. found that the number of macrophages in inflamed synovial tissue increased significantly, with both M1 and M2 macrophages increasing, but the proportion of M1 macrophages was significantly higher than M2 macrophages. If the depletion of macrophages with clodronate liposomes, synovial tissue thickening can be significantly reduced, lymphocytes and vascular penetration can be reduced, and finger protruding nodules on synovial tissue can be repaired [61]. If the M1/M2 macrophage ratio is regulated using subjective intervention, the progression of OA could be significantly suppressed. Huang et al. constructed an immunoglobulin G binding bilirubin/JPH203 self-assembled nanoparticles (NPs), which could significantly inhibit M1 polarization after intra-articular administration, subsequently inhibited the inflammatory environment and promoted cartilage repair in the OA rat model [62]. Therefore, it is not difficult to see that the uncoordinated polarization of macrophages and the increase of M1 macrophages play an extremely important role in the promotion and maintenance of synovitis. The polarization of macrophages in the M1 direction will release an abundant of inflammatory factors such as TNF- α and IL-1 β [63]. First of all, these inflammatory factors will promote synovial-like fibroblasts release more inflammatory factors, secondly, M1 macrophages will secrete a lot of NLRP3 protein, which causes synovial fibroblasts to appear excessive pyroptosis, when the pyroptotic cell content are released to extracellular, and as part of macrophage activation of DAMPs activation, prompting inflammation continued progress [64]. In addition, M1 macrophages also secrete BAFF. As one of the members of the TNF superfamily, it can activate and promote the proliferation of B cells, but also affect the number and function of T cells, and can upregulate Th1 cell-mediated inflammatory response when overexpressed [65]. CD40 is highly expressed in synovial tissue of OA patients [66] and it acts as a member of the TNF- α receptor family, mainly expressed by macrophages. Its ligand is CD40L, a member of the TNF superfamily that is mainly expressed by activated T cells. CD40/CD40L is a very typical adaptive immune signal [67]. After the combination of the two, it will initiate both classical and non-classical NF- κ B signal transduction pathways, MAPK signaling pathway, PI3K/AKT signaling pathway, and further aggravate the inflammatory response [67]. In addition, this signal combination also has a recruitment effect. Raised Th1 cells and Th 17 cells significantly increase the content of inflammatory factors such as TNF- α and IL-1 β in synovial fluid. In them, Th 17 also reacts with the surface antigen of synovial fibroblasts to form an immune response, enabling it to secrete matrix metalloproteinases (MMPs) [68].

In summary, synovitis in OA is driven by a dominant M1-polarized macrophage population, which amplifies inflammation through TNF-α, IL-1β, IL6, IL-18, IL-22, CCL2 and CXCL8 [43,55] (Fig. 3B). This imbalance suppresses M2 macrophages' reparative functions in the secretion of TGF-β, IL-4 and IL-10, creating a self-sustaining inflammatory loop [43,69] (Fig. 3C). Critically, experimental depletion of macrophages or nanoparticle-mediated M1/M2 rebalancing reduces synovitis and cartilage damage, confirming macrophages as central therapeutic targets.

As mentioned earlier, other immune cells also exist in the synovial tissue. Among them, T cells can be classified into T helper (Th) cells, cytotoxic T cells, and regulatory T cells (Tregs) based on their functions in the immune response. Th cells respond to B cells, cytotoxic T cells have an immune-killing effect after activation, and Treg cells have immunosuppressive function [70]. At present, the progression of OA is related to multiple Th cells, including Th1, Th9, Th17, which are differentiated from CD4^+^ T cells in the synovial tissue, release and transmit inflammatory factors in the pro-inflammatory response, activate macrophages and osteoclasts, stimulate chondrocytes to express matrix metalloproteinases and cause pain in patients [71]. The imbalance in the number of Th1/Th2 and Th17/Treg is the main reason for maintaining chronic inflammation in OA [6]. During the progression of OA, Th1 cells secrets pro-inflammatory factors such as interferon-γ (IFN-γ) and interleukin-2 (IL-2), while Th17 cells provide other pro-inflammatory factors including IL-17, IL-21, IL-22, IL-26, TNF-α and receptor activator of nuclear factor-κ B Ligand (RANKL) to aggregate OA [6,69] (Fig. 3B). On the contrary, those anti-inflammatory factors and phenotypes expressed by Th2 cells (IL-4, IL-5 and IL-10) and Treg cells (TGF-β, IL-35, CD69 and IL-10) to maintain the normal type of joint [6,72](Fig. 3C) are decreased when OA occurs.

#### 2.3.2 Immune cells or immune-like cells derived from bone marrow and subchondral bone effect OA

Beyond synovial tissues, immune cells or immune-like cells derived from bone marrow and subchondral bone also significantly influence OA pathogenesis. Among them, bone marrow-derived monocyte-derived macrophages and osteoclasts are notably important.

In mature bone tissue, most of the osteoclasts and macrophages originate from the same ancestor: adult bone marrow-derived HSCs that give rise to common myeloid progenitor cells (CMPs) [73]. Based on current research in mouse models, HSCs-derived monocytes function in two ways: circulating monocytes that are lowly expressed for the surface marker Ly6C in mice, and migrating monocytes that are highly expressed for Ly6C in mice [74]. The former enters the circulatory system from the bone marrow and are subject to the regulation of the immune system, while the latter is typically understood as activated monocytes because they enter the vasculature from the bone marrow and either quickly transform into circulating monocytes or penetrate the endothelium to enter tissues where they are further differentiated into other antigen-presenting cells or tissue macrophages, such as osteoclasts and giant cells [75]. Although osteoclasts and macrophages share a common ancestor, osteoclasts also have another source, namely yolk sac-derived erythro-myeloid progenitors (EMPs). This progenitor cell-derived osteoclast is typically a long-term osteoclast precursor that is attached to the subchondral bone and responsible for bone remodeling in physiological or pathological environments. EMPs and HSCs lineages are independent, but there may be cell-to-cell fusion between them [76]. This suggests that osteoclasts may play diverse roles in the bone remodeling of OA. In subchondral bone, macrophages also play a role in shaping the inflammatory microenvironment. When inflammatory factors, such as TNF-α, originating from synovial fluid or other systemic sources reach the subchondral bone, or when resident cells like bone cells and chondrocytes release inflammatory mediators and intracellular contents due to senescence or apoptosis, the local macrophage population is influenced, promoting polarization towards the M1 phenotype [77]. At this time, M1 macrophages will release TNF-α and IL-6, and when these two inflammatory factors are treated *in vitro* with human peripheral mononuclear macrophages, they will significantly promote their differentiation into osteoclasts, and up-regulate the expression levels of IL-1β, TNF-α and IL-12, further exacerbating the inflammatory microenvironment of subchondral bone [78,79]. Macrophages in subchondral bone not only regulate the inflammatory microenvironment by releasing inflammatory factors through paracrine action, but also affect bone formation by regulating the decomposition of adipose tissue in bone marrow [80]. Stimulated by mechanical movement, bone marrow macrophages overexpress reticulocalbin 2 (RCN2), which promotes bone formation by activating the cAMP-PKA signaling pathway [81]. It is not known whether this osteogenic effect is related to osteophytogenesis, but the mechanism of response to mechanical stress once again confirms the complexity of the genesis of OA. In addition, obesity significantly increases the content of white adipose tissue in the epididymis, and is accompanied by increased vascular infiltration and macrophage infiltration. Macrophages attached around blood vessels secrete osteopontin (OPN), which accumulates in bone marrow and binds with osteoclasts on the surface of bone trabeculae to promote the expression of MMP9 by osteoclasts and improve the bone resorption capacity of osteoclasts. Finally, significant decreases in bone volume fraction and bone mineral density compromise the bone's ability to withstand mechanical stress, increasing hyaline cartilage wear and thereby accelerating the progression of OA [82]. Bone is a highly dynamic organ in the human body, which is constantly changed and remodeled due to the existence of osteoclasts and osteoblasts [83]. As the organ most closely associated with hyaline cartilage, subchondral bone not only crosstalk through the paracrine pathway, but also coordinate together to offset the negative effects of mechanical stress [81]. If the dynamic balance of the bone is broken, both the increased activity of osteoblasts and the increased activity of osteoclasts will lead to the loss of normal function, which will further reveal the wear and tear of the cartilage, which will in turn feed back to the subchondral bone and accelerate the lesion of the subchondral bone [84]. The main function of both resident osteoclasts and migrating monocyte derived osteoclasts is to mediate bone resorption. Normally, osteoclasts and osteoblasts coordinate with each other through the RANK-RANKL pathway. However, as mentioned above, an inflammatory microenvironment appears in the subchondral bone, and inflammatory factors such as interferon-γ (IFN-γ), TNF-α and IL-1β secreted by macrophages can promote the differentiation of osteoclast precursors into osteoclasts, which undoubtedly enhances the bone absorption of subchondral bone [81]. This pathological bone loss exacerbates hyaluronic cartilage erosion, chondroproteoglycan loss, and increases the thickness of calcified cartilage, which can be significantly improved by the use of drugs such as Ruboxistaurin [85]. In addition, excessive bone resorption by osteoclasts can lead to changes in the microstructure of subchondral bone. The original purpose of this transformation is to adapt to better adaptive mechanical distribution, but the over-activation of osteoclasts leads to the failure of osteoblasts to play their osteoblastic role, which results in the decrease of bone density and a serious imbalance in the ratio of columnar bone microstructure to plate bone microstructure, forming many vertical pores, which provide space for the expansion of new blood vessels. It helps them gradually break through the subchondral bone boundary and penetrate into hyaline cartilage, participating in the hyaline cartilage degradation process and aggravating OA [86]. At the same time, the presence of blood vessels will change the physical and chemical properties of subchondral bone, including pressure, fluid flow, oxygen concentration and pH value, resulting in changes in subchondral bone homeostasis [87]. In addition to the subchondral bone microenvironment composed of bone cells, bone progenitor cells, osteoblasts and osteoclasts, subchondral bone also has a microenvironment in the bone marrow. Resident bone marrow MSCs (BMSCs) differentiate into osteoblasts, and B cells are responsible for regulating the local immune microenvironment [88]. On the one hand, B cells regulate the production of osteoclasts through RANKL/OPG signaling system, and the RANKL expressed by B cells is also a key protein for their own development [89]. On the other hand, when B cells receive antigens, they will differentiate into effector B cells, which have the ability to synthesize and secrete immunoglobulins [90]. Of course, not all B cells exacerbate OA, and there is also a type of B cell in the bone marrow that specializes in secreting IL-10, called regulatory B cells [90]. M1 macrophages significantly inhibited RANKL-induced osteoclastogenesis, primarily through the secretion of IL-12, rather than IL-10 [91]. T cells also play a significant role in regulating the subchondral bone microenvironment. RANKL also has an activation effect on T cells, so when osteoblasts and osteocytes overexpress RANKL, T cell progenitors will be rapidly homing to the thymus and boosted T cell production [92]. Th17 cells are one of the cells that affect RANKL content, it does not directly express it, but secret IL-17A affects all cells that can produce RANKL and then activate osteoclasts and T cells [93]. It can also up-regulate the levels of inflammatory factors such as TNF-α, IL-1β and IL-6 in the subchondral bone microenvironment [94,95]. Like B cells, T cell groups also have anti-inflammatory functions in the subchondral bone microenvironment, such as Treg cells. Treg cells can promote apoptosis of osteoclast progenitors by expressing cytotoxic T lymphocyte-associated antigen-4 (CTLA-4) and binding with CD80/CD86 on the surface of osteoclast progenitors [96]. It can also inhibit the progression of inflammation by secreting cytokines such as TGF-β and IL-10 [97](Fig. 3C).

#### 2.3.3 Synergistic destructive effect of chondral microenvironmental changes and inflammation in OA

Articular cartilage is composed of 5 % chondrocytes and 95 % of its ECM. Although there is only a single cell type, the mechanical stress, electrical signal, and physical and chemical signal subjected to articular cartilage are completely different due to their different distribution locations and depths [98,99]. Articular cartilage can therefore be divided into hyaline cartilage, which covers the surface of the joint and counteracts shear stress and friction, and fibrocartilage, which provides high tension and is located at the bone-tendon junction [100]. The functional disparity is primarily due to the varying compositions of collagen fibers within the ECM. Hyaline cartilage is characterized by a high concentration of type II collagen, whereas fibrocartilage contains a denser network of type I collagen. This distinction is largely influenced by the microenvironment surrounding chondrocytes. When this microenvironment alters, so does the functionality of the chondrocytes as well. Consequently, it has historically been postulated that the degradation of hyaline cartilage observed in OA is attributed to dysregulation of ECM remodeling by chondrocytes, a consequence of changes in the microenvironment of cartilage.

Factors affecting the cartilage microenvironment mainly include mechanical stress stimulation, oxygen concentration, cytokine changes, immune cell groups, and chondrocyte senescence induced by injury [101,102]. The main function of articular cartilage is to reduce the mechanical load generated during movement as much as possible, so that the joint can withstand more force. Because of this, frequent and unbalanced mechanical stress stimulation is the biggest culprit that causes changes in the cartilage microenvironment [82]. As described earlier, subchondral bone is constantly subjected to micro-fractures and then healed in order to withstand this unbalanced mechanical stress. This process requires the coordination between osteoblasts and osteoclasts, in which PIEZO1, a stress-sensing protein, is involved. When the expression of PIEZO1 changes, it affects the expression of collagen type II alpha 1 chain (COL2A1) in chondrocytes and the proliferation and activation of osteoclasts, thus changing the composition of the ECM of cartilage and the structure of subchondral bone [32,103]. However, the aforementioned process is predicated on the assumption that no inflammatory lesions are present in any of the components. In reality, as the ECM of cartilage is solely produced by chondrocytes, the capacity of chondrocytes to repair the cartilage ECM is significantly constrained by the influences of chondrogenic development [104]. Under such conditions, the stress necessary to support the subchondral bone progressively intensifies. In response to this increased demand, there is an augmentation of bone mass in certain regions; however, it is more common for microfractures to arise as the subchondral bone is subjected to mechanical stress [82]. There is a marked increase in the presence of macrophages at the sites of microfractures. Conversely, the depletion of macrophages at these sites can result in the downregulation of genes crucial to regeneration, such as SRY-box transcription factor-9 (SOX-9) and COL2A1 [105]. In addition, the activity of osteoclasts at the fracture site increased in order to clear the damaged ECM first, but interestingly, if this mechanical stress change occurred frequently, the fluid in the lacunar tubules would increase the cutting force on the bone cells, resulting in apoptosis of the bone cells. After apoptosis, the cell membrane would break and the contents would be released outward through the lacunar duct. At this time, if osteoclasts clear the damaged ECM of bone, they will accelerate the outward release of the contents, resulting in the expression of a large number of inflammatory factors such as IL-1β and TNF-α by neighboring osteoblasts and osteoblasts. Most importantly, they will be prompted to express RANKL, thus enhancing the membrane fusion of osteoclast precursor cells to form multinucleated osteoclasts. It also improves the survival rate of osteoclasts [106]. This imbalance eventually leads to bone loss, further reducing the mechanical stress that subchondral bone can withstand and increasing the wear and tear of chondrocytes [82]. This effect on the subchondral bone microenvironment also contributes to the immunologic regulation described above, as well as to angiogenesis and invasion of hyaline cartilage. When blood vessels invade hyaline cartilage, they will not only destroy the structure of hyaline cartilage, but also change the oxygen partial pressure and oxygen concentration of the cartilage microenvironment [107]. Articular cartilage always maintains an anoxic environment due to the absence of blood vessels [108]. But when a large number of blood vessels invade, it not only creates a new immune exchange channel for the hyaline cartilage, but also an oxygen exchange channel for it. Oxygen will bring more electron transfer to chondrocytes and naturally increase reactive oxygen species (ROS) accumulation, which provides the basis for the establishment of oxidative stress microenvironment [109,110]. In addition, these blood vessels come from bone marrow, and plasma cells and T cells in bone marrow can further act on articular cartilage [111,112].

ROS is a by-product of the redox reaction of oxygen molecules in the process of biological oxidation, and has a typical dual effect. The threshold value established by each tissue for ROS is different, but the uniform thing is that when ROS is lower than the threshold value in these tissues, it mainly acts as a multifunctional and pleiotropic physiological signal transduction agent, regulating its function through oxidative modification of proteins. However, if ROS is above this threshold, non-specific toxic effects on DNA, proteins, and lipids can cause damage to cells and genetic structure [113]. In addition, ROS plays a role in the physiological activity of T cells, again through oxidative modification of T cell effector proteins and activators [114]. When blood vessels invade hyaline cartilage, T cells will enter hyaline cartilage through blood vessels, and when they are activated by activating factors, they will consume oxygen to provide energy necessary for proliferation and activation. Take the initial differentiation of T cells into CD4^+^ T cells as an example, the energy of differentiation in this process mainly comes from aerobic glycolysis. Because the initial T cells initiate metabolic reprogramming, which in turn affects the PI3K/Akt/mTOR signaling pathway and radically alters energy metabolism [115]. This is a transition from oxidative phosphorylation to aerobic glycolysis, and the final result is that when T cells are activated in large numbers, their demand for oxygen spikes, in contrast, ROS concentrations also spike locally for a short time, which naturally increases the risk of oxidative stress [115,116]. In addition, oxygen concentration also affects the differentiation of T cells. Whether the differentiation process of Th1 cells can be started is mainly regulated by T-bet and IFN-γ, which is a very strict negative feedback regulatory ring, in which the reduced expression of either of the two factors will affect the production of Th1 cells [117]. For example, knocking out lactate dehydrogenase in primary T cells reduces aerobic glycolysis and inhibits IFN-γ expression, resulting in a decrease in Th1 cells [118]. Although oxygen concentration does not specifically inhibit or promote the proliferation and differentiation of certain types of T cells, its regulation of the activation or proliferation of T cells to obtain energy, as well as its influence on the production of ROS, to a certain extent, regulates glycolytic pathways, pentose phosphate pathway (PPP), thereby modulates the differentiation of T cells through T-cell receptor (TCR) signaling pathway [7].

In addition to rising ROS levels, immune cells such as macrophages and T cells also have an impact on chondrocytes. First, they secrete a large amount of IL-1β [119], and *in vitro* treatment of healthy chondrocytes with IL-1β can cause OA phenotype [110], up-regulated expression of matrix metalloproteinases such as MMP13, down-regulated synthesis of collagen and proteoglycan, and promote apoptosis of chondrocytes [120]. Macrophages also secrete TNF-α, which also promotes ECM degradation through the up-regulation of MMP13 and other matrix metalloproteinases [121]. Activated T cells also secrete IL-6, and injecting IL-6 into the joints of mice can induce cartilage lesions similar to OA. If the IL-6 gene is knocked out in mice, the incidence and prevalence of OA in mice are inhibited [122]. The level of IL-6 in the synovial fluid of knee joints of patients with terminal OA was also significantly higher than that of healthy patients [123]. IL-17, produced by Th17 cells can promote inflammation, induce chondrocytes to secrete MMPs to promote the degradation of chondrocyte ECM, and at the same time, IL-17 can also increase the content of NO in the articular cartilage microenvironment, which can significantly promote the aging and apoptosis of chondrocytes [124]. The inflammatory synovial tissue elicits comparable immunoregulatory effects on both macrophages and T cells, due to the presence of an exceptionally high population of T cells and a significant accumulation of macrophages within the synovial tissue of osteoarthritic joints. This unique cellular composition alters the microenvironment of the articular cartilage, exerting a multifaceted influence on the progression of OA [125].

In summary, inflammation plays a crucial role in the development and progression of OA, and its occurrence inevitably triggers the activity of the innate immune system. In OA, the occurrence of inflammation often accompanies the activation of immune cells, leading to cell apoptosis and the clearance of necrotic cells, thus playing a positive role in responding to inflammation. However, due to the complex and tightly connected structure of joint tissues, the negative feedback loops involved when immune cell function are too numerous, disrupting the low-inflammatory microenvironment. Therefore, a method using exogenous MSCs for treatment has been developed. Studies show that MSCs produce many trophic factors through paracrine pathways, including growth factors, cytokines, and chemokines. These factors can stimulate MSCs from different tissues to differentiate into chondrocytes, indicating significant potential in the repair of OA cartilage tissue. In addition to their reparative functions, MSCs also have immunomodulatory capabilities, making them capable of regulating inflammation and its resolution [23]. However, the complex mechanisms by which they exert immune regulation during treatment, as well as the regulatory effects of MSCs on macrophages and CD4^+^ T cells, remain unknown.

## 3 Immune mechanisms of MSCs in the treatment of OA

With the development of regenerative medicine, MSC-based therapy technology has gradually moved from basic research to clinical trials. MSCs can effectively repair cartilage ECM, change the damaged structure of subchondral bone, and inhibit the polarization of macrophages towards pro-inflammatory phenotype [27]. This suggests that it cannot only delay OA progression by regenerating various structures of OA joints but also cut off the vicious cycle formed between inflammation and tissue structure destruction by exerting its inherent immune regulatory role. Previously, we described the promoting role of T cells and macrophages in the development of OA, and macrophages in particular are thought to be the initial inflammatory shapers of OA. This linkage between T cells and macrophages plays an important role in the maintenance and promotion of OA. However, T cells and macrophages not only have pro-inflammatory groups, but also M2 macrophages and Treg cells can effectively inhibit the progression of OA. Therefore, a large part of the immune regulation of MSCs is achieved by regulating T cells and macrophages for OA therapy (Fig. 3).

### 3.1 Indirect immune regulation of MSCs in the treatment of OA

The development, maintenance and development of OA are never the result of a single factor, so the therapeutic effects of MSCs are not specific to a particular tissue. Nowadays, synovitis is considered to be another main factor inducing cartilage lesions. Synovial fluid can provide a wear-resistant and low-friction surface for articular cartilage, but after synovial tissue inflammation, synovial fluid can provide very limited lubrication, resulting in increased shear stress on articular cartilage [126]. Increased ECM wear in chondrocytes leads to the transformation of these matrix fragments into DAMPs that activate macrophages. As mentioned above, after the occurrence of synovitis, the number of macrophages in synovial tissue increases sharply, and after being activated by DAMPs, they become polarized toward the M1 pro-inflammatory phenotype, resulting in a series of immune lesions [127]. The use of subpatella fat pad-derived MSCs (IFP-MSCs) can effectively reverse synovitis and reduce fibrosis of synovial tissue, especially significantly improving the degradation of Substance P, a key regulator of local inflammatory and fibrotic responses. Without this inflammatory mediator, the immune imbalance in synovial tissue can be quickly repaired [128]. However, the use of MSCs therapy needs to consider whether it can stably reside and exert the aging of the paracrine pathway, because in this process, IFN-γ secreted by activated Th1 cells can induce apoptosis of MSCs through NO aggravation [129]. The wear of cartilage ECM not only activates macrophages, but also reduces the mechanical stress borne by them, resulting in dysregulation of subchondral bone immune homeostasis. Mechanical wear caused by insufficient synovial lubrication is one of the reasons, and another reason is that chondrocytes do not repair cartilage ECM in time [82]. Adipose MSCs (AD-MSCs) have the ability of autologous differentiation and the ability to inhibit the release of inflammatory mediators and avoid the overactivity of the immune system by paracrine effect [130,131]. Therefore, it has shown excellent application prospects in the treatment of OA.

Li et al. injected AD-MSCs into the joint cavity of OA rats can significantly promote chondrocytes to express chondrogenic factors such as ACAN, COL2A1 and SOX-9, and significantly inhibit the expression of matrix-degrading enzymes such as MMP13. At the same time, AD-MSCs also show strong chondrogenic differentiation ability, which can further assist chondrocytes in repairing cartilage ECM [132]. Even though BMSCs are more difficult to obtain and culture compared with AD-MSCs [133], they are similar and both can repair cartilage defects. Zhu et al. injected BMSCs loaded with hydrogel into the knee cavity of OA rats, and also observed the repair of cartilage ECM, and BMSCs also showed a strong ability to differentiate into chondrocytes [134]. The inhibition and repair effects of these MSCs on cartilage ECM degradation can make articular cartilage better bear mechanical stress and inhibit DAMPs production [2,27]. At the same time, restoring the ability of articular cartilage to withstand mechanical stress can also reduce the load of subchondral bone. Subchondral bone is the main source of pain in OA, and also the main culprit that aggravates the wear of articular cartilage [135]. The major pathological changes were myelopathy and functional incompatibility between osteoclasts and osteoblasts. As mentioned above, frequent hydromechanical changes will cause excessive apoptosis of osteocytes [106]. Under the influence of osteoclasts, intracellular substances will be released rapidly and activate macrophages and T cells, and activated T cells will further recruit mononuclear macrophages and activate osteoclasts, forming a malignant feedback [106]. In addition, extracellular vesicles (EVs) have been demonstrated as one of the main factors that maintain the functions of MSCs, and also play pivotal roles in cartilage therapy [136]. Shen et al. showed that BMSCs-Exos derived lncRNA TUC339 could significantly down-regulate TNF-α in the joint cavity of OA rats [137]. Yang et al. also obtained similar results by using exosomes from UC-MSCs [138]. TNF-α itself can promote apoptosis of bone cells, and the specific mechanism involves multiple signaling pathways. In addition, TNF-α can rapidly increase the intracellular TLR4 content of bone cells that have undergone apoptosis, and then cause necrotic apoptosis of bone cells from point to point [139]. Therefore, by applying MSCs and inhibiting osteocyte apoptosis, the inflammatory activation of macrophages can be greatly reduced. After mediating bone absorption, osteoclasts increase the pores in the subchondral bone plate and promote vascular hyperplasia, then break through and invade into the hyaline cartilage, and reveal the damage of cartilage, blood vessels will also change the oxygen concentration, and further affect the activation and recruitment of T cells and macrophages. There are two ways to use MSCs to repair subchondral bone structure. One is to use MSCs to regulate mononuclear macrophages for multi-nuclear fusion and reduce the content of osteoclasts. The other is to use MSCs to carry drugs and induce them to differentiate into osteoblasts, thus balancing the functions of osteoclasts and osteoblasts. However, in the OA microenvironment, osteoblasts will be affected by activated CD4^+^ T cells and overexpress RANKL, leading to osteoclast activation [92,140]. In addition, although osteoblasts can play the function of bone repair, how can to control the location? How is the degree of repair controlled? These are questions we need to think about in the treatment of OA. The most suitable MSCs to differentiate into osteoblasts are dental pulp MSCs (DP-MSCs) and BMSCs. Using Astragaloside-IV can significantly promote the differentiation of BMSCs into osteoblasts [141]. Lu et al. found that exosomes from BMSCs could inhibit osteoblast apoptosis by regulating the MAPK pathway [142]. Kaustubh Raundal used acellular biological scaffolds of spinach to significantly promote the adhesion, proliferation and osteogenic differentiation of DP-MSCs [143]. As mentioned above, angiogenesis has a very complex impact on the OA microenvironment, especially the establishment of oxidative stress microenvironment. The activation and differentiation of T cells require a large amount of oxygen, and ROS generated during this process will in turn become factors regulating the activation and differentiation of T cells [7]. In a mouse model of colitis, high glucose levels were used to up-regulate the ROS concentration released by mitochondria, which promoted Th17 cell differentiation by increasing TGF-β and exacerbating autoimmunity [144]. ROS not only promotes the differentiation of Th17 cells but also inhibits the differentiation of Treg cells [6]. By inhibiting the production of ROS in the process of oxidative metabolism, the content of Treg cells will be significantly increased, thus improving the psoriasis of mice [145]. The study of Liu et al. showed that AD-MSCs and synovial MSCs could significantly inhibit the production of ROS by OA chondrocytes [146]. Yao et al. found that AD-MSCs Exos could reduce the ROS content by up-regulating the NRF2 content [147].Therefore, the use of MSCs therapy can play an immune regulatory role by affecting the content of ROS on the one hand to improve the oxidative stress microenvironment and on the other hand to inhibit the activation and differentiation of T cells. And how MSCs regulate physical, chemical and energetic microenvironment in OA will be discussed later.

### 3.2 Direct immune regulation mechanism of MSCs in OA

The reason why researchers believe that the pathogenesis of OA is complex is precisely because it involves many structures and close crosstalk. A change in one structure can set off a chain reaction that contributes to OA. When applying MSCs for OA therapy, by restoring the vitality of chondrocytes, inhibiting the degradation of chondrocyte ECM, and repairing the chondrocyte ECM and subchondral bone structure, it can indirectly inhibit the activation of T cells and inhibit the activation of macrophages, so as to play a role in immune regulation. But in fact, MSCs have a direct regulatory effect on T cells and macrophages. AD-MSCs can significantly up-regulate the expression of Treg cell-related genes and inhibit the expression of Th17 and Th1 cell-related genes, and high concentration of AD-MSCs can also promote the differentiation of Th1 cells into Th2 [148]. This regulatory effect may be due to the fact that AD-MSCs up-regulate the expression of CD69 in CD4^+^ T cells. In CD69 deficient mice, the ratio of CD4+T cells differentiate into Th1 and Th17 is increased due to the transcription of RORγt and the activation of Stat3. In contrast, the CD69 induced by the antigen-specific signal negatively regulates Th17 differentiation through activating Stat5 [149]. Another research confirmed the decrease of Foxp3(+) Tregs in CD69 deficient mice [72]. As previously mentioned, the imbalance of Th17/Treg cell ratio is a major factor in the maintenance and development of chronic inflammation, and CD69 is generally regarded as a negative regulator of Th17 activation and a positive regulator of Treg cell activation [150]. UC-MSCs also have a very significant regulatory effect on Th17 cells. When co-cultured with T cells, UC-MSCs can significantly down-regulate the content of T cells RORγt and inhibit the expression of IL-23 receptors in T cells. The former is a strong transcription factor of IL-17, while the latter responds to IL-23. It is used to induce differentiation of T cells into Th17 cells [[151], [152], [153]]. Menstrual blood-derived MSCs (Men-MSCs) can significantly inhibit T cell differentiation to Th1 and Th17, most notably by reducing IFN-γ, IL-17, and GM-CSF secreted by these inflammatory T cells. In addition, it also inhibits the proliferation of CD4^+^ T with high expression of CD40L, which has unusual significance for cutting off the persistent inflammation caused by T cells [154]. MSCs demonstrate immunomodulatory properties, notably by suppressing T cell-mediated immune responses. Specifically, diverse MSC populations have been shown to inhibit the differentiation of CD4^+^ T cells into pro-inflammatory Th1 and Th17 subsets. Furthermore, MSCs promote the differentiation of immunosuppressive regulatory Tregs. These effects, coupled with the potential of MSCs to inhibit T cell recruitment and proliferation, contribute to their overall immunosuppressive capacity. After T cells are activated, especially after differentiation into inflammatory T cells, CCL5 is highly expressed, and a ligand with a high affinity is CCR5, through which macrophages can be recruited into inflammatory lesions [155]. This recruitment effect is to promote inflammation in the body and is a protective mechanism. However, it is difficult to enter the anti-inflammatory response later, so that the inflammation persists for too long, forming a complex malignant feedback mechanism with various structures of OA. IFN-γ can also activate macrophages to polarize toward M1, and the synergistically released inflammatory factors such as TNF-α and IL-1β can activate other T cells and macrophages [156]. In particular, TNF-α promotes apoptosis, so this crosstalk matrix is not only complex, but also has a very negative effect. Macrophages themselves have the function of antigen presentation and form antigen-MHC complexes by binding with the tissue–compatible complex MHC, thus activating T cells and promoting T cell differentiation [157]. In addition, Th17 cells up-regulate the content of RANKL in inflammatory sites by secreting IL-17, IL-21, IL-22, etc., thus regulating the production of osteoclasts, and even acting as the precursor of osteoclasts [158]. Treg cells directly inhibit the expression of RANKL by secreting anti-inflammatory factors such as IL-10 and TGF-β, thus inhibiting the differentiation of osteoclast precursors into mature osteoclasts [159]. In conclusion, MSCs are effective in the treatment of OA, and during this process, they play a role in immune regulation, which on the one hand directly affects T cell differentiation and regulates macrophage polarization. On the other hand, they play an indirect role in immune regulation by repairing the tissue structure associated with OA. In this process, T cells play an important mediating role. In fact, the discussion of the immune system is more about rheumatoid arthritis, but the chronic inflammation in OA is actually closely related to the immune system, so the subsequent development can use some immunosuppressive modifications to treat MSCs in advance, and then transplant them into OA, perhaps to better play the therapeutic effect of MSCs.

### 3.3 Metabolisms in OA progression and the role of MSCs on it

When OA occurs, in addition to the pathological changes of each component that constitutes the joint cavity, it will also cause various pathological and chemical property changes in the joint cavity. Firstly, the catabolism in the inflammatory area is enhanced, and the activity of oxidase in mitochondria increases. During the OA progression, a large amount of oxidative factors including ROS are produced due to glycolysis and lactate dehydrogenase A (LDHA) [160]. As inflammation progressively intensifies, a substantial release of inflammatory factors can impair mitochondrial function, leading to the inactivation of key oxidases within these organelles. Concurrently, excessive oxygen consumption during the early stages of inflammation further compromises cellular respiration, resulting in a shift toward anaerobic glycolysis as the predominant metabolic pathway in affected tissues. This metabolic alteration leads to the accumulation of significant amounts of incompletely oxidized metabolites [161]. The previous text has stated that T cell differentiation and proliferation require a significant amount of glucose [162]. However, the extremely low oxygen content in the center of the inflammatory area means that glucose can only eventually be converted into incomplete oxidation products such as lactic acid, pyruvate, and α-ketoglutaric acid, which gradually accumulate [163]. This is just the process of sugar metabolism; there are also fat metabolism and protein metabolism, and these processes will also be found to be changed in the development of OA [164]. Affected by inflammation, cells will disintegrate, and their contents will be released outside the cells. In addition, in the case of abnormal protein metabolism, intact cells will also release a large amount of peptides and free amino acids into the extracellular space [165]. These substances combined with lactic acid produced by anaerobic fermentation will rapidly lower the pH value in the inflammatory area. As the pH value decreases, a large amount of H^+^ is released into the tissue fluid, which will cause the ionization of inorganic salts in the tissue fluid to intensify, releasing more inorganic salt ions, resulting in an increase in osmotic pressure [166]. Moreover, the increase in osmotic pressure will also trigger cell disintegration, and a large amount of cell contents will be released into the tissue fluid again. Furthermore, the vascular permeability within the inflammatory area will increase, accompanied by the exudation of plasma proteins from the blood vessels, further increasing the osmotic pressure [167]. In short, although this chronic inflammation of OA does not cause significant and rapid changes in the joint cavity like acute inflammation does, precisely because of its chronic and progressive nature, once such changes occur, they are difficult to eliminate and gradually accumulated severe.

Therefore, when MSCs are injected into the joint cavity for the treatment of OA, the regenerative characteristics or immune regulatory ability of the MSCs will be affected by the OA environment, in particular the decrease in pH value and increase of lactic acid [160]. Regarding the role of lactic acid, apart from serving as a signal to regulate the proliferation or differentiation of immune cells such as T cells [168], it also affects the differentiation direction of BMSCs. After the endothelial cells in the blood transport the lactic acid produced by glycolysis to the BMSCs, it leads to the regulation of histone lactylation in the BMSCs group, and the expression of some osteogenic-related genes such as COL1A2 and Cartilage Oligomeric Matrix Protein (COMP) will be upregulated. Thus, they gradually differentiate into osteoblasts and alleviate osteoporosis [169]. However, this study did not use other types of MSCs for the research, and it has not yet been confirmed whether it is universally applicable. In fact, the avascular nature of articular cartilage inherently creates an environment of low oxygen, and when OA occurs and develops, the recruitment of a large number of immune cells further stabilizes and intensifies this low-oxygen environment. This is accompanied by lactate accumulation, H^+^ accumulation, which leads to multi-tissue acidosis [160]. Moreover, hypoxia regulation also affects the function of MSCs. Under hypoxia regulation, the expression profile of AD-MSCs changes, mitochondrial gene expression, mitochondrial protein translation, and H^+^ driven ATP synthesis are inhibited [170]. From this perspective, it reduces the further decrease in oxygen content after foreign cells are introduced into the joint cavity, and also avoids the accumulation of incomplete oxidants such as ROS caused by mitochondrial damage. To a certain extent, this influences the activity of MSCs. Since AD-MSCs originate from adipose tissue, the influence of a low-oxygen environment on them is more direct. When fat metabolism is insufficient, many fatty acids will remain in the joint cavity. These fatty acids may damage the differentiation ability of AD-MSCs [171]. However, there are also experiments that have confirmed that the exosomes secreted by hypoxia-induced AD-MSCs can effectively treat lumbar facet joint OA [172]. This indicates that the mRNA or cytokines secreted by AD-MSCs in a hypoxic environment can effectively promote cartilage repair. In summary, the hypoxic environment causes the MSCs to make corresponding adaptive changes. In the joint cavity of OA, such changes may be beneficial for the differentiation and proliferation of MSCs. Additionally, lactic acid, as a product of glycolysis, has extremely complex effects on the entire microenvironment. Recently, an energy-stimulating strategy for MSCs culture was established using an optimized culture medium to promote the mitochondria and ATP production of MSCs, thereby mitigating OA by reducing ROS [173]. Thus, modulating the metabolism and energy status of MSCs may be a future state-of-the-art.

The alteration of osmotic pressure also has a significant impact on the occurrence and development of OA [174]. Osmotic pressure has been proven to be closely related to the differentiation of stem cells. A high osmotic pressure of 400 mOsm or lower can induce AD-MSCs to differentiate into nucleus pulposus-like cells [175]. For hematopoietic stem cells, by adding glucose or sodium chloride to the culture medium to increase the osmotic pressure, the efficiency of these stem cells differentiating into natural killer cells can be enhanced by approximately ten times [176]. Furthermore, the relatively easily modifiable physicochemical property of osmotic pressure also affects the synthesis and transplantation of biological tissue engineering materials. The common cell culture system is carried out in a two-dimensional manner in a culture flask, but when combined with materials, it can achieve three-dimensional culture. Under three-dimensional (3D) culture conditions, the cell morphology is different from that in two-dimensional (2D) culture, mainly because 3D culture can provide a more comfortable living environment for the cells. When MSCs are cultured in three dimensions, after adjusting the osmotic pressure to a hypotonic condition, the MSCs will effectively undergo osteogenic differentiation, which is significantly related to the change in cell volume [177]. The homing mechanism of MSCs after transplantation into the joint cavity has not been fully elucidated yet. However, it is certain that the MSCs after transplantation do not merely adhere to the surface of the joint cartilage, but will penetrate into the defect areas of the joint cartilage. Therefore, MSCs are basically in a similar 3D culture environment after transplantation. At this time, changes in osmotic pressure are very likely to affect the differentiation of MSCs. In addition to the direct influence on MSCs, osmotic pressure also affects the biological materials carrying the cells. By constructing complexes using special materials to carry nanofats or other lubricating drugs and transferring them into the OA rat model, it can effectively repair joint lubrication and reduce joint cartilage wear [178]. This release of nanomaterials based on microfluidic technology, the rate of drug release is also determined by the osmotic pressure of the liquid medium. A high osmotic pressure will significantly slow down the release rate of the drug [179].

In summary, after being transplanted into the joint cavity of OA, MSCs will be affected by the microenvironment of the joint cavity, and their activity and stem cell-like characteristics will change. When using materials to carry and deliver MSCs, multiple complex factors need to be taken into account. At the same time, appropriate screening of the type of MSCs selected is also necessary. If drugs are carried, it is also necessary to consider whether the drugs can maintain their activity in an acidic environment with high H^+^ concentration.

### 3.4 The different immunomodulation roles between MSCs and other immune cells, as well as precautions to be taken

NK cells originate from lymphoprogenitor cells, which are derived from hematopoietic stem cells in the bone marrow [180]. The development and activation of NK cells both require regulation by cytokines, and the sites of development and maturation of different NK cells, as well as their functions after maturation, are all different. For example, CD56^bright^ NK cells exist in secondary lymphoid tissues and mature into CD56^dim^ NK cells in peripheral blood. Generally, CD56^dim^ NK cells have stronger cytotoxicity and express the CD16 receptor, while CD56^bright^ NK cells produce more cytokines but have a lower expression level of CD16 [181]. CD56^dim^ NK cells are generally regarded as a more mature form of NK cells. However, although there are some differences, they can still release immune-related factors such as IFN-γ and TNF [182]. As natural killer cells, or as immune cells, it is normal for them to exert immune functions. The reason why NK cells are the highly sought-after seed cells in adoptive immunotherapy lies in their unique "missing self" immune mechanism, and they can have killing capabilities without the need for special factors to activate them. Based on this ability, NK cells can effectively target tumors, and this is the main application direction of NK immunotherapy at present [183]. However, there are also diseases in the field of “arthritis” that can be treated with NK cell-related immunotherapy, namely rheumatoid arthritis (RA). As a joint disease caused by autoimmune deficiency, most patients need to take immunosuppressive drugs for a long time to suppress the progression of the disease [184]. However, this can lead to many complications. Therefore, it is expected in clinical practice that immunosuppressive drugs can be reduced or even avoided to control the condition. CD56^dim^ NK cells have been demonstrated as protectors in RA [185]. Recent research has provided the evidence of CD8^+^CD57^+^KIR2DL1^+^ NK cells are related with sustained remission, which is pivotal in RA therapy [186]. These data provide a screening basis for NK cell therapy for OA, but they also raise questions. During the development, maturation, and function of NK cells, there should be many subgroups. This is indeed the case. The development and maturation of NK cells are significantly influenced by the environment. Although NK cells can be activated by a single receptor such as CD16 to initiate development and differentiation, the majority of NK cell development and differentiation are regulated by the array of receptors on the cell membrane surface, and these receptors not only have activation-related receptors but also have inhibition-related receptors. Many cytokines such as TGF-β can also inhibit the activation of NK cells [187]. Although NK cells can be activated *in vitro* before transplantation, it is unknown whether they can maintain their specific phenotype after being injected into the joint cavity, or whether they can further differentiate into the NK cell subset characterized by CD8^+^CD57^+^KIR2DL1^+^ or transform into other phenotypes with slightly weaker killing ability. Additionally, NK cells are more focused on their killing effect, which seems to be contrary to the original intention of improving the inflammatory microenvironment in the joint cavity. Even if they can eliminate senescent cells, this is still the case. This is why NK cells are mainly applied in tumor treatment rather than other inflammatory-related diseases. Even for the treatment of RA, it has not yet been applied in clinical practice. Therefore, the uncertainty in their development and differentiation, as well as their overly strong immune regulatory ability, may make the application of NK cells in the treatment of OA take a relatively long time.

Dendric (DC) cells and cytokine-induced killer (CIK) cells have been clinically applied. However, recent meta-studies have shown that using DC-CIK cells instead of T cells as the seed cells for adoptive immunotherapy can lead to many side effects, including but not limited to hematological side effects, digestive system side effects and skin side effects. Hematological side effects include leukopenia and anemia; digestive system side effects include anorexia and vomiting; skin side effects include rash. Although this is closely related to the cancer cycle and the number of injected cells, these side effects far exceed the tolerance range of conventional inflammatory disease treatments [188]. Regarding the above-mentioned side effects, it is currently unclear whether they are caused by the direct killing effect of the cells or the large amount of cytokines secreted. However, this also raises thoughts. During DC-CIK cell therapy, the general way of cell infusion is intravenous injection, which can cause the cells to travel throughout the body and even pose a threat to the kidneys and liver. Whether local injection is possible is still unknown at present. However, great prognosis evidence has been shown in a study using local injection combined with intravenous infusion of CAR-T cells for the treatment of B-cell lymphoma [189]. Therefore, the best therapeutic method may be the combination of intravenous injection and local injection for not only tumor but also OA. In short, cells will eventually enter the peripheral blood and pose a threat to the kidneys and liver as well. This point is not only for NK cells, DC-CIK cells, but also MSCs. After injecting MSCs into the joint cavity, local immune enhancement will be triggered. Many immune cells are recruited from peripheral blood, activated and differentiated in the inflammatory area, and produce related phenotypes. During the onset of OA, the vascular content significantly increases, and even vascular infiltration occurs in the cartilage. Moreover, these blood vessels can deliver factors released by distal organs, such as cardiokines, adipokines and myokines, to the OA area [190]. Furthermore, a large number of anti-inflammatory factors such as IL-4 and IL-33 can cause skin barrier damage, mainly by down-regulating the expression of epidermal antibacterial proteins and several differentiation markers and 2D and 3D cultured keratinocytes [191]. Meanwhile, IL-4 can significantly promote the production of specific Immunoglobulin E (IgE). Currently, many people worldwide suffer from IgE-mediated food allergies [192]. Therefore, for such patients, whether MSCs therapy can be applied still needs further consideration. In fact, the excessive levels of IL-4 and IL-13 are not only related to food allergies, but also the pathogenesis of many allergic diseases is associated with abnormal levels of these two factors. Therefore, the impact of this local immunity on the overall immunity should not be overlooked when conducting the subsequent clinical transformation of MSCs therapy for OA.

### 3.5 Current clinical application of MSCs-based therapy for OA and future perspectives

In recent years, MSCs have been widely studied in clinical trials. It has been over 50 years since MSCs were first discovered. During this period, MSCs have been isolated from various sources such as umbilical cord, liver, placenta, and synovium, and have been tested in a large number of clinical studies. As of Jun 30th 2025, more than 500 clinical trials based on MSCs have been recorded in the National Institutes of Health's clinical trial database in the United States (https://clinicaltrials.gov/). We applied the “Osteoarthritis” as the Condition/disease and “Mesenchymal stem cell” or “Mesenchymal stromal cell” as the Intervention/treatment in the website of clinicaltrials.gov (Table 1). There were 47 “Mesenchymal stromal cell” and 134 “Mesenchymal stem cell” trials applied for OA therapy. However, there were only 9 of them that reported results and 3 of them reached Phase 4 in total. Until now, there are only two MSC drugs in the market for OA therapy, including CARTISTEM® approved by the Ministry of Food & Drug Safety (MFDS) of Korea since 2012 [21] and JointStem® approved by the Japan Health and Welfare Ministry since 2015 [193]. Comparatively, CARTISTEM® is composed of UC-MSCs with a suggested dose at 2.5 × 10^6^ cells/500 μL/cm^2^ (size of knee cartilage defects), whereas JointStem® is composed of AD-MSCs with a suggested dose at 100 million cells/patient. CARTISTEM® is recommended for the treatment of repetitive and/or traumatic cartilage degeneration, including degenerative osteoarthritis characterized by an ICRS grade IV cartilage defect, without any age restrictions [21]. The indications for JointStem® are based on criteria that include patients aged between 18 and 75 years who have idiopathic osteoarthritis of the knee graded as 2 or higher according to the Kellgren–Lawrence classification. Additionally, these patients must have experienced an average pain intensity of grade 4 or higher on a 10-point visual analog scale (VAS) for a minimum duration of 4 months [194]. However, both of the two were not market approved in other countries.

**Table 1:** MSC trials for OA therapy found in clinicaltrials.gov.

Subjects		Mesenchymal stromal cell	Mesenchymal stem cell
Study Phase	Early Phase 1	2	6
	Phase 1	22	55
	Phase 2	19	62
	Phase 3	4	15
	Phase 4	2	1
	Not applicable	7	30
Study Status	Not yet recruiting	4	5
	Recruiting	8	13
	Active, not recruiting	4	9
	Completed	17	55
	Terminated	1	3
Study Results	With results	2	7
	Without results	45	127

In recent years, extracellular vesicles (EVs) have emerged as a key focus of research, particularly in facilitating the repair and regeneration of cartilage, among which MSC-derived EVs are most popular [136]. Regardless of intravenous injection, subcutaneous injection or other administration methods, MSC-Exos are excreted from the body within a short period of time. Therefore, discussing how to prolong the half-life of exosomes may have a positive impact on the local therapeutic effect of MSCs-Exos [195]. In addition, MSCs are often used in combination with biomaterials, scaffolds, and even nanoparticles. Hydrogels can target drugs to specific sites and control drug release for cartilage regeneration [196]. Zhang et al. found that loading MSC-Exos onto chitosan hydrogel as a carrier can improve the stability of proteins and miRNAs in MSC-Exos and extend their retention time in the body [197]. Additionally, they coupled cartilage-targeting peptides to the surface of MSCs to prolong their residence time in the mouse knee joint cavity, accelerating the cartilage regeneration of the damaged cartilage explants in OA patients [198]. Other genetic engineering strategies such as modifying chimaeric antigen receptors (CARs) on MSCs to enhance the immunomodulation effects [199] have also enhanced confidence in the clinical applications of MSCs.

Although MSCs therapy has made significant progress over the past few decades, there are still many challenges to be overcome. The main challenges include immunocompatibility, stability, heterogeneity, differentiation, and migration ability. More and more studies are concentrating on attempts to overcome these shortcomings. The specific mechanism of the immunomodulatory effect of MSCs is still unclear, any attempts to improve the efficacy of MSCs still lack evidence. However, preclinical studies are developing rapidly, and more standardized clinical trials are being widely conducted. It can be expected that the transition to formalized registered MSCs therapies will thrive over time. The lessons learned from current MSCs research may provide key guidance for researchers in pursuing further translational processes. With the clarification of MSCs' effectors and the emergence of new technologies facilitating in-depth research, MSCs are expected to be proven as effective treatment options for various degenerative diseases.

## 4 Conclusion

The pathogenesis of OA is complex, especially immune cells play a complex regulatory role in the maintenance and development of OA. MSCs exert immune regulatory effects through two main mechanisms. Firstly, they directly affect the immune cell population by inhibiting M1 macrophage polarization while promoting M2 macrophage differentiation, as well as inhibiting the proliferation and differentiation of pro-inflammatory T cells. Secondly, MSCs promote the repair of pathological tissues related to OA, cutting off multiple negative feedback pathways during the pathogenesis of OA, thereby fundamentally inhibiting the occurrence and further deterioration of inflammatory reactions [200]. However, the immune system mediated inflammatory response is necessary for clearing harmful cells in the body, initiating tissue damage repair, and maintaining normal tissue function [201]. Especially in recent years, research on adaptive immunity has shown more clearly how immune cells help the body avoid the invasion of infectious and malignant diseases through delicate reactions [202]. In this case, should the powerful immune regulatory mechanism of MSCs injected into the joint cavity affect the immune response of other parts of the body, thereby causing side effects? Except for using bioengineered materials to anchor MSCs at the worn area of articular cartilage, any direct injection of MSCs cannot guarantee stable homing reactions and long-term survival in the microenvironment of the joint cavity [203]. In the joint cavity of OA, severe inflammatory and oxidative stress microenvironments can induce MSCs to exhibit both inflammatory and aging phenotypes. During this process, the released inflammatory factors, contents or cell debris after apoptosis can also activate the inflammatory response of macrophages and T cells. This not only fails to treat OA, but also exacerbates it. Of course, this does not mean that targeted implantation of MSCs is absolutely safe. The anti-inflammatory factors released by MSCs can spread throughout the body through blood vessels and lymphatic vessels under synovial tissue, as well as blood vessels and lymphatic vessels in subchondral bone, which may be harmful to other local tissues undergoing normal immune responses in the body. In short, MSCs can treat OA by selectively differentiating into chondrocytes, filling cartilage defects, and reducing oxidative stress. These advantages undoubtedly prove that MSCs are indeed the most promising drugs for treating OA. However, its powerful immune regulatory ability is a double-edged sword, so in the clinical stage, it is necessary to consider related issues such as application dosage, transplantation method, and treatment cycle.

In addition, OA as a chronic inflammation caused by multiple factors, can also be a concurrent inflammation with specificity. It is worth considering whether to prioritize inflammation suppression or tissue repair in clinical treatment, and different MSCs transplantation strategies need to be developed according to different patients. Moreover, if priority is needed to improve the inflammatory microenvironment of the joint cavity, the use of MSCs transplantation therapy may not be the preferred choice, as it cannot ensure the activity and dryness of MSCs in such harmful environments. In contrast, the use of anti-inflammatory drugs or NK cell transplantation may have a more significant therapeutic effect. At present, research on the transplantation of NK cells for disease treatment is gradually becoming more abundant, and it has been confirmed that NK cells can indeed play a strong immune regulatory role and improve the inflammatory microenvironment. However, when using them, the negative impact of NK cells on immune regulation also needs to be considered [204,205].

In summary, MSCs have strong immune regulatory effects and can effectively treat bone and joint diseases by directly or indirectly regulating macrophages and T cells, play an indispensable mediating role in this process [206]. However, despite significant progress in the treatment of OA with MSCs, there are still challenges such as the heterogeneities of macrophages and T cells, low survival rate of MSCs, and individualized treatment needs [207]. For example, there are significant differences in the subpopulations of synovial macrophages among different OA patients, with M1 type dominating in some patients and M2 type dominating in others. Similar phenomenon has been raised in the multi-differentiation of T cells during OA. This heterogeneity may lead to inconsistent therapeutic effects of MSCs. There are significant differences in the levels of inflammatory and pro-apoptotic factors in the joint cavity between severe OA patients and mild OA patients [208], which in turn have a significant impact on the activity and stemness of MSCs after transplantation. Therefore, in the future, it is necessary to develop patient stratification strategies, such as detecting macrophage and T cell phenotypes through synovial fluid or determining whether MSCs are suitable through joint effusion, in order to design the optimal treatment plan.

## Data availability

No data was used for the research described in the article.

## Declaration of generative AI and AI-assisted technologies in the writing process

During the preparation of this work the author(s) used ChatGPT-4o in order to improve readability. After using this tool/service, the author(s) reviewed and edited the content as needed and take(s) full responsibility for the content of the publication.

## Declaration of competing interest

The authors declare the following financial interests/personal relationships which may be considered as potential competing interests:Juan Wang reports financial support was provided by Hainan Yize Biotechnology Co., Ltd. However, her role as a project leader does not influence the independence of this research. The entire study was conducted independently of any involvement from the company. The company did not have any influence on the design, data collection, analysis, or interpretation of the reviews. All conclusions drawn in this review are solely based on the research literature, and there is no conflict of interest in relation to the content of this paper. If there are other authors, they declare that they have no known competing financial interests or personal relationships that could have appeared to influence the work reported in this paper.
